# Depressive symptom prevalence after intracerebral hemorrhage: a multi-center study

**DOI:** 10.1186/s41687-018-0083-0

**Published:** 2018-11-23

**Authors:** Brandon A. Francis, Jennifer Beaumont, Matthew B. Maas, Eric M. Liotta, David Cella, Shyam Prabhakaran, Jane Holl, Abel Kho, Andrew M. Naidech

**Affiliations:** 1 Hauenstein Neurosciences, Mercy Health Saint Mary’s, 220 Cherry St, Grand Rapids, MI 49503 USA; 2 Institute for Public Health and Medicine, Hauenstein Neurosciences, Northwestern University, 233 Erie St, Chicago, Il 60611 USA; 3 Department of Medical Social Sciences, Hauenstein Neurosciences, Northwestern University, 233 Erie St, Chicago, Il 60611 USA

**Keywords:** Intracerebral hemorrhage, Quality of life, Depression, Neurocritical care, Antidepressant medication

## Abstract

**Introduction:**

Depressive symptoms in patients with intracerebral hemorrhage (ICH) are common and are associated with worse outcomes. It is not well described how often depressive symptoms are ascertained and treated in large unselected cohorts of patients, and whether depressive symptoms would be a potential target for improving outcomes.

**Methods:**

Data were electronically retrieved from a multi-center EHR repository in Chicago, IL, from 2006 to 2012 (“multicenter cohort”). In the multicenter cohort, we retrieved diagnostic codes and medication data from four university health systems across Chicago. In the single center cohort, we prospectively screened for depressive symptoms (NIH Patient Reported Outcomes Measurement Information System, PROMIS, T Score ≥ 60), at one, three and twelve months after ICH onset. It should be noted that not all depressive symptoms are optimally characterized through diagnostic codes.

**Results:**

Diagnostic codes for depressive symptoms up to three months after ICH onset were recorded in 132 of 3422 (3.8%) of the multicenter cohort; fewer than 10% of patients received a typical medication to treat depressive symptoms, and < 2% one month later. In the single-center cohort, PROMIS assessments were indicative of depressive symptoms in 26 of 116 (22.4%), and depressive symptoms were more likely to be found with screening (OR 7.20, 95% CI 4.5–11.5, *P* < 0.0001). Results were similar up to 12 months after ICH.

**Conclusions:**

Depressive symptoms in patients with ICH are more common than medication treatment or a coded diagnosis in a multi-center cohort, and are a potential opportunity for additional treatment to improve outcomes. There are currently no AHA/ASA treatment guidelines for depression screening of patients with ICH.

## Background

Intracerebral hemorrhage (ICH), bleeding into brain tissue, has a high morbidity and mortality [1, 2]. Since there are limited treatment options that reduce mortality in patients with ICH [3], attention has turned to optimizing health-related quality of life (HRQoL) outcomes, although there are few known ways to do so.

Depressive symptoms are common, important to patients and caregivers, impact the delivery of other health services, and reduce HRQoL. In patients with ischemic stroke, depression has been documented in almost 25% of patients [4] and up to one third of patients with any type of stroke [5, 6]. Further, depressive symptoms are readily treatable, and antidepressant treatment may confer benefits beyond mood symptoms. Fluoxetine, a selective serotonin reuptake inhibitor (SSRI), may improve depression scores in patients with ischemic stroke [7], although the long-term efficacy has not been clearly demonstrated [8].Antidepressant treatment following stroke has also been shown to decrease mortality in a placebo-controlled trial [9], underscoring the importance of screening and treating eligible patients. This has not been demonstrated in patients with ICH, for whom there is no treatment approved by the US Food and Drug Administration.

Depressive symptoms after ICH were found in approximately 20% of patients in a clinical trial using the Hamilton Depression Rating Scale (HDRS) and the Beck Depression Inventory-II (BDI-II) [10, 11] and have been associated with lower HRQoL [12]. Under-recognition of depressive symptoms could be an impediment to treatments aimed at improving HRQoL after ICH. It is not known how often depressive symptoms are documented in a representative sample of ICH survivors, and whether this would be a target for improving outcomes in a general cohort similar to what has been demonstrated in ischemic stroke.

We tested the hypothesis that depressive symptoms are less frequently documented and treated than the incidence of depressive symptoms would justify in patients with ICH. This study may be provide insight into considering antidepressant treatment as a potential opportunity to improve HRQoL in survivors of ICH.

## Methods

To determine the prevalence of depressive symptoms after ICH across the metropolitan area (“multicenter cohort”), we utilized the Chicago HealthLNK Data Repository (HealthLNK). The logistics, procedures and patient privacy issues have been described elsewhere [13]. Briefly, HealthLNK is a health data exchange consisting of merged and de-duplicated patient electronic health records (EHRs) from institutions across the Chicago area. HealthLNK includes demographic and clinical visit data from five major academic centers (Loyola University Medical Center, Northwestern Memorial, Rush University Medical Center, University of Chicago Medical Center, and University of Illinois Hospital & Health Sciences System), a large county healthcare system (Cook County Health and Hospital Systems), and a network of community health centers (Alliance of Chicago). Unique patient IDs are created for patients in HealthLNK from their demographic data using a HIPAA compliant Secure Hash Algorithm 512 hashing algorithm, allowing merging of patient data across sites without sharing protected health information. De-duplication and merging data from multiple sites creates a more accurate and complete overall record of patient care, and accounts for diagnoses and procedures completed at more than one institution. For this investigation, diagnostic and medication data were available from four institutions (Loyola, Rush, University of Chicago, and Northwestern) from 2007 to 2012, but the source of data for each patient was not discoverable.

We identified patients with the diagnostic code 431 (Intracerebral hemorrhage) from the International Classification of Diseases, 9th Ed (ICD-9). We retrieved all the other diagnostic codes for the cohort from any institution who participated in HealthLNK and searched the list for any diagnostic codes that could reasonably indicate depressive symptoms. We excluded patients who were also associated with ICD9 codes for traumatic brain injury, since these patients might have an intracranial hematoma as a complication rather than the primary diagnosis. We queried this cohort of patients for commonly used SSRIs and tricyclic antidepressants (TCAs) (e.g., sertraline, venlafaxine, fluoxetine, citalopram, escitalopram, fluvoxamine, paroxetine, imipramine, amitriptyline). Diagnostic codes and medications were associated with a specified month and year.

Separately, we prospectively identified consecutive patients with spontaneous ICH in the Northwestern University Brain Attack Registry (NUBAR) as part of an ongoing registry from January 2011 through January 2015 (“single center cohort”) who would have HRQoL follow-up data. All patients had a diagnosis of spontaneous ICH confirmed by a board-certified neurologist with head computed tomography (CT). Patients with trauma, hemorrhagic conversion of ischemic stroke, or structural lesions (e.g., tumor) were excluded. We approached patients or a legally authorized representative during the index hospitalization and asked for written consent to track identifiers and obtain outcomes, a preferred telephone number and email addresses. The study was approved by the Northwestern University Institutional Review Board. Our methods for obtaining HRQoL with the NIH Patient Reported Outcomes Measurement Information System (PROMIS) have been previously described [14] and has been validated against the gold-standard (interview) [15]. We obtained HRQoL at one, three and twelve months follow-up by sending an email with a link to complete the HRQoL assessment, the usual method. Respondents could also answer HRQoL questions over the telephone with study staff performing proxy entry, recording answers on behalf of a patient or family member. We administered computer adaptive banks [15] for the PROMIS Depression instrument. Computer adaptive testing algorithms ask questions at the predicted level of HRQoL until further data are unlikely to alter the estimate. Results are expressed in T scores, continuous numbers where the general US population scores 50 ± 10.

We defined a positive screen for depressive symptoms at a T Score of 60 or greater, one standard deviation above the US population mean, which has been validated against other standard measures such as the Beck Depression Inventory-II (BDI-II), Center for Epidemiologic Studies (CES) – Depression and other standard assessments [16]. Due to staggered follow up, some patients were only captured at 3 month and/or at the one year time-point.

Further information on the algorithms, underlying iterative response theory and detailed information about these and other available instruments is available at www.nihpromis.org.

The same information was not available from each database due to different rules and requirements that were a part of initial database development. Additionally, each database was set up as a research-only process. Clinicians did not have access to Neuro-QoL or prescription data. Clinicians could not have identified the participants in the database to inform treatment decisions. It was not permissible to cross-reference patients between NUBAR and HealthLNK as per confidentiality agreements.

## Results

**Fig. 1:**
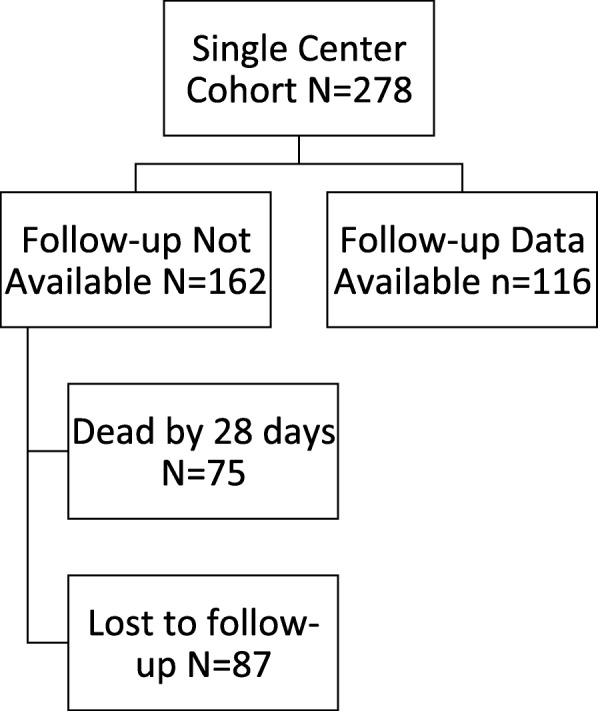
Ascertainment of patients in the single center study

Demographics of the sample are shown in Table 1. Of 3422 patients in the multicenter cohort, 1777 (51.9%) were male with a mean age of 57.7 years (+/− 15.9) and most were Caucasian (1464, 42.7%). The prospective cohort consisted of 278 patients with ICH. Demographics were similar between the multicenter and prospective cohorts except the prospective cohort was slightly older (62.5 ± 14.5 versus 57.7 ± 15.9 years, *P* = 0.001). Figure 1 shows the patient flow through the study.

**Table 1:** Demographics of the 3422 patients in the Multi-Center cohort

Variable	N (%) or Mean ± SD
Race	
1 – Native American	16 (0.4)
2 - Asian	104 (3)
3 - Black	1280 (37.4)
4 - Hispanic or Latino	94 (2.7)
5 - Pacific Islander	5 (0.1)
6 - White	1464 (42.7)
7 - Declined	74 (2)
8 – Other	91 (2.7)
Missing	294 (9)
Ethnicity	
1 – Hispanic or Latino	318 (9)
2 - Not Hispanic or Latino	2736 (79.9)
Missing	368 (10.8)
Insurance	
1 – Medicare	1488 (43.5)
2 – Medicaid	326 (9)
3 – Private Insurance	1006 (29.4)
4 – Self-pay	201 (6)
5 – No Charge	14 (0.4)
6 – Other	65 (2)
Missing	322 (9)
Sex	
1 – MALE	1777 (51.9)
2 – FEMALE	1638 (47.9)
Missing	7 (0.2)
Age, years	57.7 ± 15.9

No depressive symptoms were documented in the multicenter cohort prior to ICH diagnosis. Code 311/311.0 (depressive disorder) was the only code indicative of depressive symptoms found in the list of diagnoses for the multicenter cohort, which are shown in Table 2. Diagnostic codes for depressive symptoms up to three months after ICH onset were recorded in 132 of 3422 (3.8%) of the multicenter cohort, versus 26 of 116 (22.4%) in the prospectively screened cohort (OR 7.20, 95% CI 4.5–11.5, *P* < 0.0001). Results were similar considering depressive symptoms up to 12 months after ICH, 140 of 3422 patients in the multicenter (4.0%) versus 36 of 116 prospective patients (31.0%, OR 10.55, 95% CI 6.87–16.2, P < 0.0001).

**Table 2:** Frequency of Diagnostic Codes in the Multicenter Cohort

Diagnosis	ICD-9 Diagnostic Code	Frequency (data are N)
Intracerebral hemorrhage	431	6689
Subarachnoid Hemorrhage	430	270
Pneumonia	486	217
Depressive Disorder	311	209
COPD	496	115
Old Myocardial Infarction	412	98
Stroke	436	89
Phlebitis	451	48
Pulmonary Congestion	514	46
Acute Liver Necrosis	570	39
Malignant Prostate Neoplasm	185	37
Contusion Face/Scalp	920	31
Post Inflammatory Pulmonary Fibrosis	515	25
Phlebitis Intracranial Sinus	325	22
Sarcoid	135	21
Other Acute Ischemic Heart Disease	411	21
Renal Failure NOS	586	14
Other Severe Malnutrition	262	11
Hydronephrosis	591	11
Disease of Nail	703	10

The multicenter cohort includes all patients with ICH but does not specify the reason a patient could not be assessed for depressive symptoms such as death or a neurologically devastated state or any other possible etiology for inability to be assessed. After accounting for this difference between the cohorts by adjusting the single center cohort to include all patients with ICH, regardless of rates of ascertainment for follow up, results were similar (OR 6.07, 95% CI 2.36–5.14, P < 0.0001).

Since depressive symptoms might be treated without a specific diagnostic code recorded in the medical record we also examined administrations of antidepressant medications (Table 3). In the multicenter cohort < 10% of patients received an antidepressant medication within three months of ICH onset, with nearly all treatment within a month of ICH onset. TCAs were used in < 1% of the multicenter cohort. In the multicenter cohort, a diagnostic code for depressive symptoms was associated with increased odds of receiving an antidepressant medication (OR 8.9; 95% CI 6.34–12.54; *P* < 0.001). Among patients with a diagnostic code for depressive symptoms, 72 of 150 (48%) received an antidepressant medication within three months, while in patients without a diagnostic code for depressive symptoms, 307 of 3272 (9.4%) received an antidepressant within three months (*P* < 0.00001). There were no codes for anxiety identified in either cohort.

**Table 3:** Anti-depressant medication administration in the Multi-Center cohort. Only medications found in at least 0.5% of the cohort are shown (see Methods)

Medication	Calendar Months After ICH Onset			
	0 Months	1 Month	2 Months	3 Months
Citalopram	126 (3.4)	14 (0.4)	17 (0.5)	27 (0.7)
Sertraline	110 (3)	24 (0.7)	17 (0.5)	12 (0.4)
Fluoxetine	39 (1)	7 (0.2)	3 (0.09)	2 (0.06)
Venlafaxine	26 (0.7)	5 (0.1)	3 (0.09)	2 (0.06)
Paroxetine	30 (0.9)	4 (0.1)	2 (0.06)	1 (0.03)
Data are N (%)				

## Conclusion

We found that diagnostic codes for depressive symptoms across a metropolitan area in patients with ICH were uncommon despite a high prevalence of depressive symptoms in a representative prospectively identified cohort at one of the participating institutions. When ascertained, the prevalence of depressive symptoms was in line with a large, international clinical trial of patients with spontaneous ICH [9], underscoring that depressive symptoms are common in the general population of patients with ICH, and suggest our findings are generalizable. Screening for depressive symptoms may be reasonable because they are associated with worse outcomes, although it is not part of the current American Heart Association/American Stroke Association ICH treatment guidelines [11]. Since antidepressant medication may impact outcomes in patients with acute ischemic stroke [7], proactive depression screening and antidepressant medication may be a rational strategy to improve HRQoL in patients with ICH, a morbid disease without any specific approved therapy [8, 17–19].

We found that the rates of antidepressant treatment were < 10% the month of ICH diagnosis, and < 2% thereafter. The index hospital stay may be the most feasible time to screen for early depressive symptoms because patients are already in the hospital and it is known that early depressive symptoms are common after ICH [6]. Screening during the hospitalization may account for the finding that the large majority of prescriptions for antidepressant medications were in the calendar month of ICH onset. The Diagnostic and Statistical Manual of Mental Disorders, 5th Edition (DSM-V) requires at least a two-week period of symptoms that are consistent with the diagnosis of depression in the absence of medications or medical conditions that could better account for the symptoms, however, making diagnosis shortly after ICH onset problematic. Had we only considered codes for depressive symptoms in the calendar months after ICH onset that meet the DSM-V time criteria the incidence of diagnostic codes for depressive symptoms would have been lower. In the prospective cohort, we screened at standard intervals, which likely resulted in detecting symptoms that are more enduring, and may have allowed sufficient time for depressive symptoms to develop. An approach combining both in-hospital and post-discharge screening might have increased sensitivity.

While some evidence suggests that treating depression after ICH, particularly with SSRIs, could improve the odds of good functional outcome [2–5], the possible effects of SSRIs on rates of recurrent ICH raise a need for prospective confirmatory evidence and further study. Since aspects of HRQoL beyond mobility may be of importance to patients, depressive symptoms after ICH are most appropriately investigated independently of mobility outcomes, as has been done in other clinical trials of patients with ICH [9, 20, 21]. Similarly, specific domains of HRQoL other than mobility may be of interest to patients and could be influenced by depression, such as social functioning (e.g., planning a meal with friends and family out of the routine, completing work important to the patient) and cognitive function (e.g., managing one’s own finances) [22–25].

Depressive symptoms and anxiety symptoms often overlap, and anxiety might also be treated with SSRIs. In the widely used Euro-QOL 5D instrument used in other studies of ICH [25, 26], depression and anxiety are considered together as a single domain of HRQoL. We found no diagnostic codes related to anxiety.

There are limitations to these data. The same information was not available in each database and due to requirements for the protection of health information, it was not possible for clinicians to identify the patients in the study. It is possible that patients in the multicenter cohort sought care post-ICH from another institution that is not part of HealthLNK. However, studies have evaluated this possibility and found patient migration to be an unlikely source of bias. In another large sample of 228,151 unique patients in the metropolitan area, only 2% of patients had fragmented care, underscoring that patients are likely to receive their care at a single institution, particularly for life-threatening conditions such as ICH [27, 28].

Not all possible depressive symptoms are accounted for in ICD-9 codes, so the detection of depressive symptoms is likely to be underestimated. It is possible that patients from our center have a higher prevalence of depressive symptoms, but this is unlikely as depressive symptoms are not specific to an institution and our results are similar to other cohorts that were screened for depressive symptoms. It was not possible to ascertain the number of patients that were recommended for or participated in psychotherapy as an alternative to medication to address any depressive symptoms. That could cause an underestimation of the prevalence of depressive symptoms. However, given many insurance providers require a “code-able” diagnosis to cover psychotherapy, it is likely that any patient recommended for psychotherapy would have had a diagnostic code for depressive symptoms. HRQoL data may not be representative of the entire multicenter cohort because, as we have noted elsewhere, patients with devastating ICH are less likely to have HRQoL outcomes assessed [29, 30]. Mortality data have not been readily linked to HealthLNK (the multicenter cohort), making it difficult to ascertain what proportion of patients with ICH would be alive to be screened for depressive symptoms. Even so, we found similar results including patients who died in the single center cohort, underscoring the robustness of the finding. We openly acknowledge that there are challenges in interpreting data from a large city-wide dataset with respect to screening for depressive symptoms given there is no psychiatric interview (the gold standard for detecting depressive symptoms). However, our data suggest that more intentional screening, as performed in the prospectively screened single-center cohort, is possible. The data in the literature suggest that identifying and treating depressive symptoms may be of importance to patients and caregivers and may improve outcomes [12, 24, 25, 31–34]. This may suggest that it is reasonable to consider intentional screening for depressive symptoms in patients with ICH, although there are currently no ASA/AHA guidelines to support this approach.

In sum, we found that depressive symptoms are common in survivors of ICH and are likely to be under-diagnosed and under-treated in a multicenter cohort. Treating depressive symptoms may represent a rational strategy to improve HRQoL outcomes in patients with ICH. The limitations of this study highlight the challenge in studying large datasets obtained from different databases. We recommend caution when interpreting the data reported here. More research is needed to confirm these data and provide insights into methods for optimally addressing any disparities in diagnosis and potential for therapeutic interventions for improving HRQoL.

## Abbreviations

BDI-II: Beck Depression Inventory II
CES: Center for Epidemiologic Studies
CT: Computed tomography
DSM-V: The Diagnostic and Statistical Manual of Mental Disorders, 5th Edition
EHR: Electronic health records
HealthLNK: Health link data repository
HRQoL: Health related quality of life
ICD-9: International classification of diseases 9th edition
ICH: Intracerebral hemorrhage
NIH: National Institutes of Health
NUBAR: Northwestern University Brain Attack Registry
PROMIS: Patient reported outcomes measurement information system
SSRI: Selective serotonin reuptake inhibitor
TCA: Tricyclic antidepressant

## References

[1] Bhalla A, Wang Y, Rudd A, Wolfe CD (2013). Differences in outcome and predictors between ischemic and intracerebral hemorrhage: The South London stroke register. Stroke.

[2] Feigin VL, Lawes CM, Bennett DA, Barker-Collo SL, Parag V (2009). Worldwide stroke incidence and early case fatality reported in 56 population-based studies: A systematic review. Lancet Neurol.

[3] Morgenstern LB, Hemphill JC, Anderson C, Becker K, Broderick JP, Connolly ES (2010). Guidelines for the management of spontaneous intracerebral hemorrhage. A guideline for healthcare professionals from the american heart association/american stroke association. Stroke.

[4] Broomfield NM, Quinn TJ, Abdul-Rahim AH, Walters MR, Evans JJ (2014). Depression and anxiety symptoms post-stroke/tia: Prevalence and associations in cross-sectional data from a regional stroke registry. BMC Neurol.

[5] Hackett ML, Yapa C, Parag V, Anderson CS (2005). Frequency of depression after stroke: A systematic review of observational studies. Stroke.

[6] Karamchandani RR, Vahidy F, Bajgur S, Vu KY, Choi HA, Hamilton RK (2015). Early depression screening is feasible in hospitalized stroke patients. PLoS One.

[7] Chollet F, Tardy J, Albucher JF, Thalamas C, Berard E, Lamy C (2011). Fluoxetine for motor recovery after acute ischaemic stroke (flame): A randomised placebo-controlled trial. Lancet Neurol.

[8] Hackett ML, Anderson CS, House AO (2005). Management of depression after stroke: A systematic review of pharmacological therapies. Stroke.

[9] Jorge RE, Robinson RG, Arndt S, Starkstein S (2003). Mortality and poststroke depression: A placebo-controlled trial of antidepressants. Am J Psychiatry.

[10] Christensen MC, Mayer SA, Ferran JM, Kissela B (2009). Depressed mood after intracerebral hemorrhage: The fast trial. Cerebrovasc Dis.

[11] Koivunen RJ, Harno H, Tatlisumak T, Putaala J (2015). Depression, anxiety, and cognitive functioning after intracerebral hemorrhage. Acta Neurol Scand.

[12] Rådholm K, Arima H, Lindley RI, Wang J, Tzourio C, Robinson T (2015). Older age is a strong predictor for poor outcome in intracerebral haemorrhage: The interact2 study. Age Ageing.

[13] Kho AN, Cashy JP, Jackson KL, Pah AR, Goel S, Boehnke J (2015). Design and implementation of a privacy preserving electronic health record linkage tool in Chicago. J Am Med Inform Assoc.

[14] Cella D, Lai JS, Nowinski CJ, Victorson D, Peterman A, Miller D (2012). Neuro-qol: Brief measures of health-related quality of life for clinical research in neurology. Neurology.

[15] Choi SW, Reise SP, Pilkonis PA, Hays RD, Cella D (2010). Efficiency of static and computer adaptive short forms compared to full-length measures of depressive symptoms. Qual Life Res.

[16] Choi SW, Schalet B, Cook KF, Cella D (2014). Establishing a common metric for depressive symptoms: Linking the bdi-ii, ces-d, and phq-9 to promis depression. Psychol Assess.

[17] Sacco S, Marini C, Toni D, Olivieri L, Carolei A (2009). Incidence and 10-year survival of intracerebral hemorrhage in a population-based registry. Stroke.

[18] Mikami K, Jorge RE, Adams HP, Davis PH, Leira EC, Jang M (2011). Effect of antidepressants on the course of disability following stroke. Am J Geriatr Psychiatry.

[19] Aarts N, Akoudad S, Noordam R, Hofman A, Ikram MA, Stricker BH (2014). Inhibition of serotonin reuptake by antidepressants and cerebral microbleeds in the general population. Stroke.

[20] Anderson CS, Heeley E, Huang Y, Wang J, Stapf C, Delcourt C (2013). Rapid blood-pressure lowering in patients with acute intracerebral hemorrhage. N Engl J Med.

[21] Chemerinski E, Robinson RG, Kosier JT (2001). Improved recovery in activities of daily living associated with remission of poststroke depression. Stroke.

[22] Ried LD, Jia H, Feng H, Cameon R, Wang X, Tueth M (2011). Selective serotonin reuptake inhibitor treatment and depression are associated with poststroke mortality. Ann Pharmacother.

[23] Löppönen P, Tetri S, Juvela S, Huhtakangas J, Saloheimo P, Bode MK (2014). Association between warfarin combined with serotonin-modulating antidepressants and increased case fatality in primary intracerebral hemorrhage: A population-based study. J Neurosurg.

[24] Hackam DG, Mrkobrada M (2012). Selective serotonin reuptake inhibitors and brain hemorrhage: A meta-analysis. Neurology.

[25] Marquez-Romero JM, Arauz A, Ruiz-Sandoval JL, Cruz-Estrada EL, Huerta-Franco MR, Aguayo-Leytte G (2013). Fluoxetine for motor recovery after acute intracerebral hemorrhage (fmrich): Study protocol for a randomized, double-blind, placebo-controlled, multicenter trial. Trials.

[26] Liotta EM, Garg RK, Temes RE, John S, Lee VH, Bleck TP (2012). Warfarin-associated intracerebral hemorrhage is inadequately treated at community emergency departments. Stroke.

[27] Galanter W, Applebaum A, Boddipalli V, Kho A, Lin M, Meltzer D (2013). Migration of patients between five urban teaching hospitals in Chicago. J Med Syst.

[28] Liotta EM, Singh M, Kosteva AR, Beaumont JL, Guth JC, Bauer RM (2013). Predictors of 30-day readmission after intracerebral hemorrhage: A single-center approach for identifying potentially modifiable associations with readmission. Crit Care Med.

[29] Naidech AM, Beaumont JL, Berman M, Liotta E, Maas MB, Prabhakaran S (2014). Web-based assessment of outcomes after subarachnoid and intracerebral hemorrhage: A new patient centered option for outcomes assessment. Neurocrit Care.

[30] Naidech AM, Beaumont JL, Rosenberg NF, Maas MB, Kosteva AR, Ault ML (2013). Intracerebral hemorrhage and delirium symptoms: Length of stay, function and quality of life in a 114-patient cohort. Am J Respir Crit Care Med.

[31] Naidech AM, Beaumont JL, Berman M, Francis B, Liotta E, Maas MB (2015). Dichotomous “good outcome” indicates mobility more than cognitive or social quality of life. Crit Care Med.

[32] Sinyor D, Amato P, Kaloupek DG, Becker R, Goldenberg M, Coopersmith H (1986). Post-stroke depression: Relationships to functional impairment, coping strategies, and rehabilitation outcome. Stroke.

[33] Gainotti G, Antonucci G, Marra C, Paolucci S (2001). Relation between depression after stroke, antidepressant therapy, and functional recovery. J Neurol Neurosurg Psychiatry..

[34] Pohjasvaara T, Vataja R, Leppävuori A, Kaste M, Erkinjuntti T (2001). Depression is an independent predictor of poor long-term functional outcome post-stroke. Eur J Neurol.

